# Avoiding early diagnosis

**DOI:** 10.1002/jgf2.70015

**Published:** 2025-04-24

**Authors:** Takao Wakabayashi, Goh Keng Wee, Mikinosuke Ishibashi, Yoshiki Akiyama, Naoki Kanda, Tomoyuki Watanabe

**Affiliations:** ^1^ Department of General and Emergency Medicine Japan Community Health‐Care Organization Sapporo Hokushin Hospital Sapporo Japan

**Keywords:** cognitive bias, diagnostic error, Lemierre's syndrome, relative bradycardia

## 1 CASE PRESENTATION

A previously healthy 27‐year‐old woman was referred from a nearby clinic for the treatment of pneumonia.

*The patient was referred with a diagnosis of community‐acquired pneumonia (CAP). Hospitalization for CAP is generally indicated when the oxygen saturation on room air falls to ≤93%, or when severity scores such as a Pneumonia Severity Index score of ≥III or a CURB‐65 score of ≥1 are present.*

Five days prior to referral, the patient visited her primary care physician with complaints of high fever, nasal discharge, and pharyngitis. Although a rapid influenza test was negative, baloxavir marboxil was administered based on the local outbreak situation. However, her fever persisted, and she revisited the clinic 1 day before referral. Blood tests and chest computed tomography (CT) were performed, leading to a diagnosis of pneumonia. She received an outpatient dose of 2 g of ceftriaxone and was prescribed levofloxacin at 500 mg/day. The next day, marked inflammatory markers were revealed in blood tests, prompting her referral.

Administering antibiotics without obtaining various cultures for infections such as pneumonia is not recommended. Additionally, initiating treatment with fluoroquinolones, such as levofloxacin, at the first visit is also discouraged. This is because fluoroquinolones have some efficacy against mycobacteria, including tuberculosis, which can delay the diagnosis of these conditions.

The patient reported intermittent high‐grade fevers, sore throat, pleuritic chest pain, and fatigue. They reported no rash, cough, vomiting, constipation, diarrhea, genitourinary symptoms, or weakness. They had previously had sinusitis. She did not take prescription medications, smoke, drink alcohol, or use drugs. She was not sexually active. She had no recent exposures to sick contacts, hot springs, travel history, undercooked meats, or bites from insects or animals.

This review of systems highlights multisystem involvement and eliminates several potentially relevant exposures. The presence of fevers and fatigue points toward infectious, inflammatory, or malignant processes.

On physical examination, her consciousness was alert, the patient was febrile; her axillary temperature was 38.0°C, the heart rate was 110 beats per minute, the blood pressure was 99/50 mm Hg, the respiratory rate was 20 per minute, and the oxygen saturation was 97% while the patient was breathing ambient air. The body‐mass index (BMI; the weight in kilograms divided by the square of the height in meters) was 19.9. Cardiopulmonary examinations revealed that holo crackles were auscultated in the right lower lung field, and a systolic murmur (Levine grade 3/6) was heard at the apex. No signs of arthritis or skin abnormalities were shown. The patient's mood, attention, and affect were appropriate.

If this case is confirmed to be pneumonia, hospitalization is indicated. While both the PSI and CURB‐65 scores are low, and oxygen saturation is maintained, the shock index (SI)—calculated as heart rate divided by systolic blood pressure—exceeds 1.0. Generally, an SI above 0.9 predicts a severe condition. Although SI is more predictive in elderly patients, it cannot be overlooked in younger individuals. Moreover, the presence of holo crackles on auscultation aligns with early findings in pneumonia. However, it would be premature to definitively diagnose pneumonia based solely on these findings. I consider the heart murmur auscultated in this case to be functional, given that the patient is in a hyperdynamic state. However, for the evaluation of fever of unknown origin, I recommend performing a transthoracic echocardiogram as an initial assessment.

The patient's laboratory results revealed a white blood cell count of 15,440 per microliter (normal range: 3,500–9,700) and an absolute neutrophil count of 13,100 per microliter (normal range: 1,120–7,189). Hemoglobin levels were 12.8 g/dL, with a mean corpuscular volume of 86.2 fl, and the platelet count was 30,000 cells per microliter (normal range: 140,000‐379,000 c). Creatinine was 0.83 mg/dL (normal range: 0.46‐0.82). Serum albumin and electrolyte levels, including calcium and phosphate, were within normal limits, as were the liver function tests. Urinalysis revealed 1+ protein and 3+ blood. The D‐dimer level was elevated at 2.49 μg/mL (normal range: 0.00–1.00). Both the international normalized ratio (INR) and fibrinogen levels were normal. The C‐reactive protein (CRP) level was markedly elevated at 34.14 mg/dL (normal value: ≤0.5).

This case presents with remarkably elevated inflammatory markers, which are findings typically uncommon in autoimmune diseases. Considering the acute onset, I suspect an infectious etiology. Additionally, thrombocytopenia is observed, raising the possibility of disseminated intravascular coagulation (DIC). Treatment for DIC caused by infection usually involves addressing the underlying disease. However, heparin administration may be considered in some cases. This case demonstrates markedly elevated inflammatory markers for typical community‐acquired pneumonia, warranting further investigation in addition to treatment.

**FIGURE 1:**
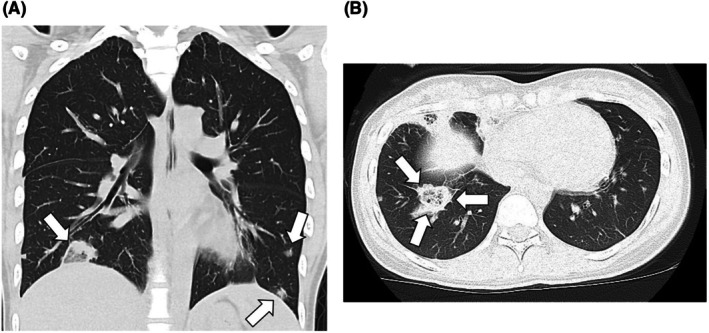
Computed tomography at admission. (A) Multiple infiltrative and nodular shadows were observed along the pleura of both lungs. (B) A reversed halo sign was noted in the right middle and lower lung fields.

The chest X‐ray taken at the time of admission showed a positive silhouette sign of the right hemidiaphragm. Since there were no images provided by the previous physician, a chest CT was reexamined just to be sure, and multiple infiltrative and nodular shadows were observed along the pleura of both lungs (Figure 1A). Additionally, a reversed halo sign was noted in the right middle and lower lung fields (Figure 1B). Based on this, treatment and further analysis were started considering not only simple bacterial pneumonia but also septic emboli. Furthermore, the CT reading suggested interstitial pneumonia with an organizing pneumonia (OP) pattern.

We initially started an assessment with the assumption of community‐acquired pneumonia, but it seems that this may not be the case. High‐resolution CT revealed a finding where a density increasing of more than 2 mm in thickness, forming a crescent or ring‐like shape around the central ground‐glass opacities, was observed. This finding is defined as the reversed halo sign and has been considered characteristic of cryptogenic organizing pneumonia (COP). However, similar findings have also been reported in cases of chronic eosinophilic pneumonia and sarcoidosis. In this case, the presence of multiple nodular shadows beneath the pleura, along with the patient's age and mode of onset, suggests, as the presenter pointed out, the possibility of septic emboli. In cases where the diagnosis is unclear, we must listen sincerely to the patient's voice. In other words, the medical interview is crucial, and we should take a history and findings that do not match the typical presentation of pneumonia. I would like to explore the possibility of a focus of infection outside the lungs with septic emboli in mind. If no such focus is found, I would like to collect cells using bronchoscopy.

After admission, blood and sputum cultures were taken for pathogen identification, and treatment with ABPC/SBT 3 g × 4/day and danaparoid sodium 1250 units × 2/day was started for pneumonia. The test results submitted at the time of admission as part of the evaluation for fever of unknown origin have been revealed. Rheumatoid factor, antinuclear antibody, CCP antibody, MPO‐ANCA, and PR3‐ANCA were all negative. Additionally, complement levels were elevated.

Since complement levels are elevated and the antinuclear antibody is negative, the possibility of systemic lupus erythematosus (SLE) is low. MPO‐ANCA is elevated in conditions such as microscopic polyangiitis, allergic granulomatosis and angiitis (Churg‐Strauss syndrome), pauci‐immune necrotizing crescentic glomerulonephritis, systemic sclerosis, and Goodpasture syndrome. PR3‐ANCA is elevated in conditions like Wegener's granulomatosis. Therefore, the pretest probability for these diseases is reduced. An increase in complement levels indicates nonspecific inflammation. A decrease in complement levels can sometimes be useful for diagnosis.

**FIGURE 2:**
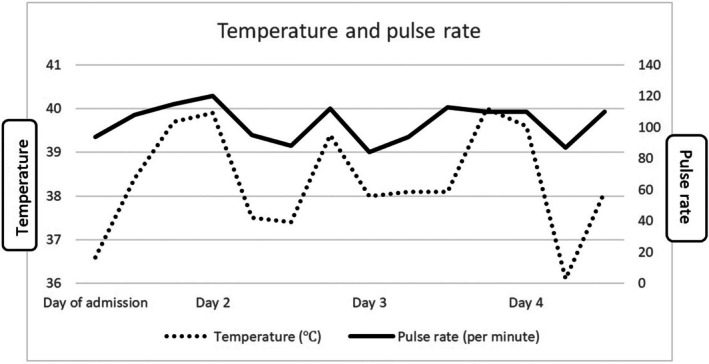
Temperature and pulse rate. A dissociation between temperature and pulse was observed.

After admission, the patient's vital signs showed relative bradycardia (Figure 2).

Under febrile conditions, an increase in body temperature beyond 38.3°C is typically accompanied by a rise in heart rate of approximately 8–10 beats per minute. This relationship, historically known as Liebermeister's rule, was first documented by Carl von Liebermeister in the late 19th century. Relative bradycardia, also known as Faget's sign, is characterized by a blunted tachycardic response to fever. Relative bradycardia is frequently observed in infections caused by intracellular pathogens, including Legionella species and *Salmonella typhi*. It has also been associated with conditions such as central nervous system lesions, lymphoma, drug‐induced fever, and adult‐onset Still's disease. As a result, relative bradycardia is regarded as a nonspecific yet clinically significant indicator that may aid in differentiating infectious from noninfectious causes at the bedside. Notably, there are no electrolyte imbalances or liver dysfunction present, and the likelihood of Legionella infection is low. However, Legionella should be considered in the differential diagnosis and checked using urinary antigen tests and other methods. Moreover, while a transthoracic echocardiogram does not definitively rule out infective endocarditis, a transthoracic echocardiogram should be performed. Some infections, such as Q fever, can cause endocarditis and are associated with relative bradycardia.

*Previously, I emphasized the importance of considering the patient's perspective. Among the patient's reported symptoms, are there any discrepancies that might impact the diagnosis of pneumonia? For instance, has the sore throat the patient mentioned resolved? What do the oropharyngeal findings suggest? The presence of relative bradycardia may certainly narrow the differential diagnosis. However, urinary Legionella antigen testing and antibody testing for autoimmune diseases require time. We need to search for the origin of fever without being overly concerned about the relative bradycardia. We should act based on the information reported by the patient.*

**FIGURE 3:**
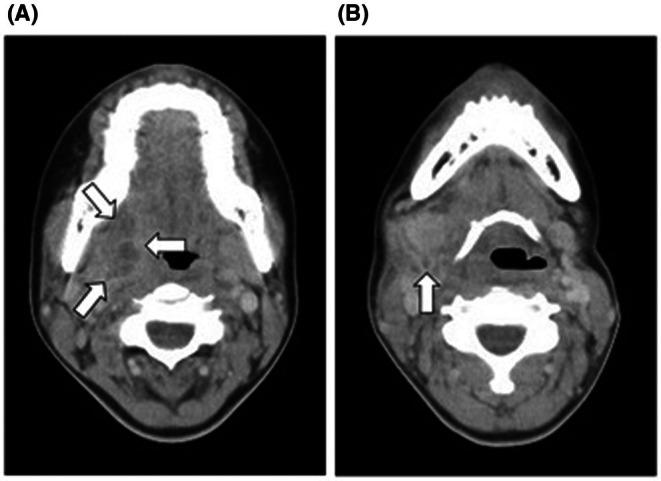
Computed tomography on the third day. (A) Low‐density area with peripheral contrast enhancement was observed. (B) A contrast defect was observed in the branch of the right internal jugular vein leading to the abscess, suggesting the presence of an intravascular thrombus.

We considered the possibility of *Legionella pneumonia* and autoimmune diseases focusing on the presence of a relative bradycardia. Autoimmune diseases such as vasculitis were considered in the differential diagnosis, and various autoantibodies were collected. Urinary Legionella antigen was negative. On the second day of admission, infective endocarditis was suspected, and a transthoracic echocardiogram was performed, but no abnormal findings were observed. Since the patient had been complaining of a sore throat since admission, on the third day of admission, a contrast‐enhanced CT of the neck was performed, which suggested a peritonsillar abscess (Figure 3A) and possible thrombosis in the internal jugular vein branches (Figure 3B). Based on this, we diagnosed the patient with Lemierre's syndrome caused by a peritonsillar abscess. The findings on the chest CT were interpreted as septic emboli. On the fourth day of admission, we consulted otolaryngology for a tonsillar puncture, but were advised to transfer the patient to a higher level medical facility, and the patient was transferred. After transfer, the patient underwent tonsillectomy and abscess drainage. Antibiotic treatment was continued. Consultations with cardiology and otolaryngology were conducted, and anticoagulant therapy was not administered. The patient was discharged without sequelae. Additionally, the autoimmune antibodies collected on the second hospital day were all negative.

The diagnosis was Lemierre's syndrome with septic emboli caused by a peritonsillar abscess. This diagnosis explains the CT findings. In most cases, necrotic cavitary lesions because of septic pulmonary embolism are observed. This case was transferred to a higher level medical facility. Lemierre's syndrome is a severe condition that can progress to brain abscesses and carries risks of long‐term sequelae and death. Therefore, I believe the decision to transfer the patient was appropriate.

## 2 COMMENTARY

Lemierre's syndrome does not have a universally established definition, but it is generally described as septic thrombophlebitis of the internal jugular vein.^1^ This condition typically originates from an oropharyngeal infection and frequently involves inflammation of the venous wall, infected thrombus formation within the lumen, inflammation of the surrounding soft tissue, persistent bacteremia, and septic embolism. In this case, Lemierre's syndrome was caused by sepsis secondary to a peritonsillar abscess. Upon reexamination after the diagnosis, no significant dental caries or other notable oral findings were observed.

**FIGURE 4:**
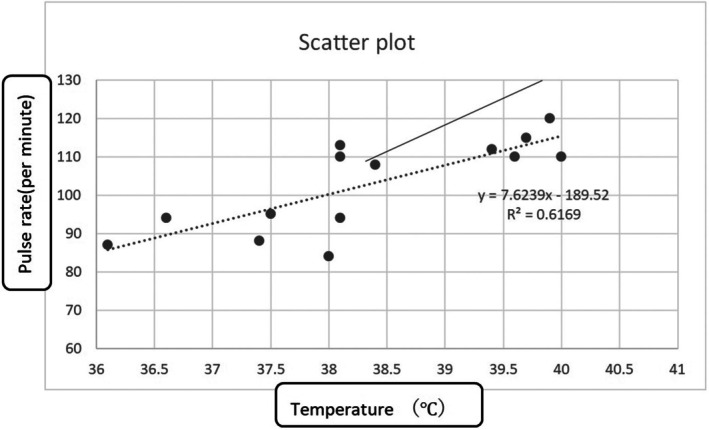
Scatterplot. The patient's vital signs show a pulse rate lower than expected for the fever. The dashed line represents the approximate regression line derived from the patient's vital signs; the solid line represents the line typically predicted.

A scatterplot created using the patient's vital signs is shown (Figure 4). The dashed line represents the approximate regression line, while the solid line indicates the predicted line typically observed. The approximate regression line lies below the predicted line, suggesting that the pulse rate increases by 7.6 beats per minute for every 1°C rise in body temperature. Therefore, the patient can be considered to exhibit relative bradycardia. Using resources such as PubMed, we searched for reports linking Lemierre's syndrome and relative bradycardia but were unable to find any. While it is reasonable to hypothesize that the causative pathogen may have contributed to the bradycardia, the specific pathogen in this case could not be identified. It is regrettable that antibiotics were administered by the referring physician without obtaining cultures, as intracellular or anaerobic bacteria might have played a role. The most common pathogen associated with Lemierre's syndrome, *Fusobacterium necrophorum* (*F. necrophorum*), is an anaerobic, nonmotile, filamentous, nonspore‐forming gram‐negative bacillus, though it is not an intracellular organism. Furthermore, no literature was found linking *F. necrophorum* to relative bradycardia.

The recommended duration of antibiotic therapy for Lemierre's syndrome is at least 4 weeks, including a minimum of 2 weeks of intravenous treatment.^1^ The duration should be adjusted based on complications such as lung abscesses or septic arthritis. When abscesses are involved, drainage in addition to antibiotic therapy is crucial. Interestingly, the rise of antimicrobial stewardship programs has led to a reduction in antibiotic prescriptions for upper respiratory infections. Paradoxically, this has contributed to a resurgence of Lemierre's syndrome, a condition once considered a “forgotten” infection.^2^ Furthermore, the effectiveness of anticoagulant therapy for Lemierre's syndrome has not been established.

In this case, it took 3 days to reach a diagnosis. The patient consistently reported pharyngeal pain, but further investigation was delayed. Several cognitive biases^3^ contributed to this delay. First, the referring physician diagnosed the patient with “pneumonia” and struggled to move away from that initial diagnosis, a phenomenon known as anchoring bias, where one becomes fixated on the initial impression. Additionally, the presence of pulmonary infiltrates on CT scans generally supports a diagnosis of pneumonia, leading to premature closure, where critical thinking halts after the initial diagnosis. This also led to confirmation bias, in which information inconsistent with the initial hypothesis was undervalued. In fact, the patient's complaints of pharyngeal pain were overlooked in this case.

These errors are not solely issues of knowledge or technical skill but are significantly influenced by systemic factors, such as chronic workload pressures. In 2015, the US Institute of Medicine released a report titled Improving Diagnosis in Healthcare, highlighting diagnostic errors as a critical healthcare challenge and emphasizing the need for collaboration among individuals, patients, and organizations to address this issue.^4^ Nishizaki et al.^5^ reported that Japanese residents had poorer knowledge regarding diagnostic errors compared with their US counterparts. It is important to recognize the substantial regional differences in knowledge and education regarding diagnostic errors. Moreover, reliance solely on clinicians' experience for diagnosis should be avoided, and the importance of education on diagnostic errors in achieving accurate diagnoses must be acknowledged.

This case highlights how an uncommon condition may present in the guise of a common disease, often complicating the diagnostic process and delaying appropriate treatment. What is the key takeaway from this case? Clinicians must pause and reconsider when they encounter subtle inconsistencies in a patient's presentation or response to treatment. Developing novel problem‐solving strategies tailored to each case is essential, as is listening attentively to the patient's voice, which often holds critical diagnostic clues.

## AUTHOR CONTRIBUTIONS

**Takao Wakabayashi:** Conceptualization; investigation; methodology; validation; visualization; writing – original draft. **Goh Keng Wee:** Visualization; investigation; methodology; writing – review and editing. **Mikinosuke Ishibashi:** Methodology; investigation. **Yoshiki Akiyama:** Methodology; investigation. **Naoki Kanda:** Investigation; methodology. **Tomoyuki Watanabe:** Methodology.

## FUNDING INFORMATION

This research received no specific grant from any funding agency in the public, commercial, or not‐for‐profit sectors.

## CONFLICT OF INTEREST STATEMENT

The authors declare that there are no conflicts of interest.

## ETHICS STATEMENT

This report was conducted in accordance of the ethical standards of the Declaration of Helsinki. Personal information of the patients was protected, and all data were recorded anonymously.

## PATIENT CONSENT STATEMENT

We provided the patient with sufficient informed consent and obtained agreement for anonymous publication. The consent was documented and stored in the electronic medical record.
